# Four new approaches for validation of Ayurvedic herbal drugs

**DOI:** 10.4103/0974-7788.72483

**Published:** 2010

**Authors:** R. D. Lele

**Affiliations:** *Head, Nuclear Medicine, Lilavati Hospital, 102, Buena Vista, JB Road, Churchgate, Mumbai 400001 E-mail: nuclearmedicinedept@gmail.com*

## INTRODUCTION

On 25^th^ May 2009 at the Homi Bhabha Birthday Centennial lecture at BARC in Mumbai, I pleaded for the urgent need to apply radiotracer technology for the validation of Ayurvedic Herbal drugs. Every new molecule introduced in modern therapeutics is first labeled with C-14 to study its absorption, biodistribution and excretion in small animals. The only tritium labeling effort of a Chinese herbal drug, Ginseng, was published in French in 1992. My new project, with financial support from DST and DBT, has been launched in collaboration with Board of Research in Isotope Technology (BRIT). Tritium labeling of Arjun bark, an important Ayurvedic drug, has been successfully accomplished and its biodistribution in small animals is being studied. Feeding C–14 urea as a manure to plants will be used as a novel approach for radiolabeling of various parts of the plant – bark, leaves, flowers, etc. and the C-14 radiolabeled plant parts will be fed to small animals and whole body autoradiography[1] will be performed to study the biodistribution of the herbal drugs. To begin with, 40 single drugs described by *Vaghbhata* will be taken for study.

## MEDHYA RASAYANAS

Ayurveda has described 10 herbal drugs as *Medhya Rasayanas - Amalaki, Ashwagandha, Bramhi, Jatamansi, Jyotishmati, Kavach Beej, Mandukparni, Shankhapushpi, Vacha*, and *Yashtimadhu*.

A transgenic mouse model of Alzheimer’s disease has now become commercially available. At birth, these animals are absolutely normal. Within 3 months, they develop changes like amyloid plaque deposition, amyloid angiopathy, Tau protein, loss of acetylcholine neurons, hypometabolism and hypoperfusion in parieto-occipital regions, etc. and the animals die within the next 6 months.

All these changes can be noninvasively shown by small animal positron emission tomography/computed tomography (PET/CT) scan,[1] with optical imaging, without sacrificing the animals. This facility is now available at ACTREC, New Mumbai, where a research project has been approved for studying the effect of the 10 *Medhya rasayanas* – singly and in combinations in 40 mice: for the first 3 months to assess the preventive potential and for the next 6 months to assess curative potential. At present, there is no effective treatment for Alzheimer’s disease. Yet, US $15 billion are spent annually on its treatment. If my study provides validation of *Medhya Rasayans* for prevention of Alzheimer’s disease, a world market of US $15 billion will be available to India.

## BIOAVAILABILITY OF AYURVEDIC HERBAL DRUGS

Bioavailability of Ayurvedic herbal drugs is a totally neglected subject. Devasagayam’s group at BARC used the inverted loop of rat intestine to study the intestinal absorption of *Terminalia arjuna* extracts as well as the active principle baicalein. Almost 15% of the baicalein (4 mg/ml) was recovered from the serosal surface, as monitored by HPLC. Both aqueous and methanolic extracts of *T. arjuna* were absorbed. I have established this facility where 40 single herbs described by *Vagbhat* will be studied for absorption (jejunum, ileum, colon). This “blind spot” in herbal drug research is frustrating for clinicians who wish to translate the laboratory *in vitro* data to clinical application. The poor bioavailabilities of oral curcumin and resveratrol are important illustrative examples.[2,3]

## HIGH THROUGHPUT SCREENING FOR MECHANISM OF ACTION

In my Haffkine Oration 2009, I have listed over 90 classes of receptors, transporters, ion channels and intracellular signaling molecules into component subtypes and subgroups, selective agonists, selective antagonists and specific radioligands of choice for mechanism-based screening of Ayurvedic drugs.

As an illustrative example, Sukh Dev (1992),[4] in collaboration with a group in USA, studied Triphala using I-125 cholecystokinin (CCK) as ligand and mouse pancreatic membrane as receptor. They showed affinity of three Ayurvedic herbal extracts – *Terminalia chebula* (96% ligand displacement), *Terminalia bellerica* (91%) and *Phyllanthus emblica* (76%), showing that “Triphala” constituents act on CCK receptors. It is surprising that in the last two decades, no further efforts were made in this direction. I have launched a DBT-supported project of mechanism-based screening of the 40 single drugs described by *Vagbhata*.

Charak states: “A single drug may have many applications owing to its diverse actions just as a man is able to perform various actions”. Many popular Ayurvedic drugs such as *Ashwagandha, Bramhi, Guduchi, Katuka, Shatavari*, etc. have multifarious properties ascribed to them. Obviously, their molecular targets are shared by many cell systems and cell membrane components such as phospholipase A2, phospholipase C, adenylyl cyclase and cAMP, adenosine receptors, eicosanoids, ion channels and neuroreceptors dopamine, serotonin, norepinephrine (NE), gamma-aminobutyric acid (GABA), etc. Stress-activated protein kinase (SAPK2) is an enzyme highly activated by bacterial lipopolysaccharides and cytokines. Many Ayurvedic Rasayan drugs act by blocking this enzyme and prevent downstream activation of NF-kB. Interestingly, NF-kB pathway activation is common to both inflammation and cancer.

Dahanukar and Thatte[5] made pioneering contribution by showing immunomodulatory action of Amlaki, *Ashwagandha, Guduchi, Haritaki, Pipalli* and *Shatavari*, all of which are now shown to suppress NF-kB activation, and regulate chronic dysregulated NF-kB pathway. Curcumin and ginger have been studied extensively to elucidate their action at the molecular level.

## CHEMOPREVENTION: FUTURE APPROACH IN MEDICINE

Chemoprevention of infection (tuberculosis, viral infection including HIV), malignancy, neurodegenerative disorders (Alzheimer’s, Parkinson’s disease), metabolic syndromes (diabetes, hypertension, atherosclerosis) should be the major focus of future Ayurvedic drug research. Devasagayam’s group at BARC has shown effectiveness of 12 Ayurvedic antioxidants at various levels – prevention of radical formation, breaking chain initiation and propagation, scavenging of primary and secondary radicals, repair of lipid membrane, repair of DNA and other cellular constituents.[6] By similar scientific validation of all other Ayurvedic drugs, apart from health benefits, a large share of global drug market will flow to India. Current global herbal market of US $70 billion is growing annually at 10–15%; global nutraceutical market is US $142 billion.

What we need is vision and imaginative thinking and swift action, as outlined, to capture our share in this huge market.
